# Supplementary material for: Borrelia miyamotoi a neglected tick-borne relapsing fever spirochete in Thailand
Source: PLoS Negl Trop Dis. 2023 Feb 21;17(2):e0011159. doi: 10.1371/journal.pntd.0011159 (PMC9983830; doi:10.1371/journal.pntd.0011159)
Supplement: S1 Table — (PDF) [file pntd.0011159.s001.pdf]

S1 Table. *Borrelia* spp. detection using a genus-based TaqMan real-time PCR assay targeting the *Borrelia* 16S rRNA gene in human samples received from Phop Phra hospital, Phop Phra district, Tak province, Thailand.

Sub-district	Number of human samples	Female	Male	Number of *Borrelia*-positive sample
**Others**	3	2	1	0
**Chong Khaep**	8	4	4	0
**Khiri Rat**	24	12	12	0
**Phop Phra**	22	8	14	0
**Ruam Thai Phatthana**	12	7	5	0
**Wale**	15	6	9	0
**Grand Total**	**84**	**39**	**45**	**0**

Note: Samples were collected during 2018-2019.
