# Supplementary material for: Borrelia miyamotoi a neglected tick-borne relapsing fever spirochete in Thailand
Source: PLoS Negl Trop Dis. 2023 Feb 21;17(2):e0011159. doi: 10.1371/journal.pntd.0011159 (PMC9983830; doi:10.1371/journal.pntd.0011159)
Supplement: Attachment — Submitted filename: Response Letter_05Jan2023.pdf [file pntd.0011159.s003.pdf]

January 05, 2023

Wen-Ping Guo
Mathieu Picardeau
Academic & Section Editors,
PLOS Neglected Tropical Diseases

Dear Professors Wen-Ping Guo & Mathieu Picardeau:

We would like to submit a revised version of the manuscript entitled "*Borrelia miyamotoi* a neglected tick-borne relapsing fever spirochete in Thailand" for consideration by the PLOS Neglected Tropical Diseases. Below you will see our responses to the reviewers' comments/questions. The revised manuscript uploaded separately shows those changes to the manuscript made in response to the reviewers' recommendations in a tracked changes and with yellow highlight file as well as a cleaned version of manuscript. In addition, the Result section has been revised to include the cross-reactivity of the in-house ELISA assay to vector-borne diseases previously reported in Thailand (Table 3) and the result description under the subtitle "Rodent exposure rate to *B. miyamotoi* and evidence of serological reactivity in humans". Moreover, the results of the serological reactivity of human sera to *Borrelia miyamotoi* recombinant GlpQ protein has been formatted into the Table 5 and the original Figure 5b and 5c were deleted from the revised manuscript. The author summary has been revised following the guidelines. This manuscript is not currently being submitted to another journal.

Thank you once again for your time and consideration.

Sincerely,

Ratree Takhampunya, Ph.D.
Department of Entomology
Armed forces Research Institute of Medical Sciences
U.S. Army Medical Component
3I5/6 Rajvithi Road
Bangkok 10400, THAILAND
Ratreet.fsn@afrims.org

The response to reviewers' comments/suggestions as the following:

1. P4, Line 3 – References are needed on this sentence such as Platonov et al. 2011 and many others.

   RESPONSE: References below were added into the manuscript as suggested;

   - Platonov AE, Karan LS, Kolyasnikova NM, Makhneva NA, Toporkova MG, Maleev VV, Fish D, Krause PJ. Humans infected with relapsing fever spirochete *Borrelia miyamotoi*, Russia. Emerg Infect Dis. 2011 Oct;17(10)
   - Gugliotta JL, Goethert HK, Berardi VP, Telford SR., 3rd Meningoencephalitis from *Borrelia miyamotoi* in an immunocompromised patient. *New Engl J Med.* 2013;368:240–245.
   - Krause PJ, Narasimhan S, Wormser GP, Rollend L, Fikrig E, Lepore T, Barbour A, Fish D. Human *Borrelia miyamotoi* Infection in the United States. New Engl J Med. 2013; 368:291–293. [PubMed: 23323920]
   - Krause PJ, Narasimhan S, Wormser GP, Barbour AG, Platonov AE, Brancato J, Lepore T, Dardick K, Mamula M, Rollend L, Steeves TK, Diuk-Wasser M, Usmani-Brown S, Williamson P, Sarksyan DS, Fikrig E, Fish D. *Borrelia miyamotoi* sensu lato seroreactivity and seroprevalence in the northeastern United States. Emerg Infect Dis. 2014; 20:1183–1190. [PubMed: 24960072]
   - Hovius JW, de Wever B, Sohne M, Brouwer MC, Coumou J, Wagemakers A, Oei A, Knol H, Narasimhan S, Hodiamont CJ, Jahfari S, Pals ST, Horlings HM, Fikrig E, Sprong H, Oers MHJ. A case of meningoencephalitis by the relapsing fever spirochaete *Borrelia miyamotoi* in Europe. Lancet. 2013; 382:658. [PubMed: 23953389]
   - Chowdri HR MD, Gugliotta JL, Berardi VP, Goethert HK ScD, Molloy PJ, Sterling SL, Telford SL III. *Borrelia miyamotoi* infection presenting as human granulocytic anaplasmosis: a case report. Ann Intern Med. 2013; 159:217.
   - Sato K, Takano A, Konnai S, Nakao M, Ito T, Koyama K, Kaneko M, Ohnishi M, Kawabata H. Human infections with *Borrelia miyamotoi*, Japan. Emerg Infect Dis. 2014; 20:1391–1393. [PubMed: 25061761]
   - Jahfari S, Herremans T, Platonov AE, Kuiper H, Karan LS, Vasilieva O, Koopmans MPG, Hovius JWR, Sprong H. High seroprevalence of *Borrelia miyamotoi* antibodies in forestry workers and individuals suspected of human granulocytic anaplasmosis in the Netherlands. New Microbes New Infect. 2014; 2:144–149. [PubMed: 25356364]
   - Jiang BG, Jia N, Jiang JF, Zheng YC, Chu YL, Jiang RR, Wang YW, Liu HB, Wei R, Zhang WH, Li Y, Xu XW, Ye JL, Yao NN, Liu XJ, Huo QB, Sun Y, Song JL, Liu W, Cao WC. *Borrelia miyamotoi* Infections in Humans and Ticks, Northeastern China. Emerg Infect Dis. 2018 Feb;24(2):236-241.

2. P4 Line 3-4 – Review articles were cited. Please cite original research articles such as Scoles et al. 2001 and Breuner et al. 2017 for *I. scapularis*, van Duijvendijk et al. 2016 for *I. ricinus*.

   RESPONSE: The below original publications were cited as recommended.

   - Scoles GA, Papero M, Beati L, Fish D. A relapsing fever group spirochete transmitted by *Ixodes scapularis* ticks. Vector Borne Zoonotic Dis. 2001 Spring;1(1):21-34.
   - Breuner NE, Dolan MC, Replogle AJ, Sexton C, Hojgaard A, Boegler KA, Clark RJ, Eisen L. Transmission of *Borrelia miyamotoi* sensu lato relapsing fever group spirochetes in relation

to duration of attachment by *Ixodes scapularis* nymphs. Ticks Tick Borne Dis. 2017 Aug;8(5):677-681.

- van Duijvendijk G, Coipan C, Wagemakers A, Fonville M, Ersöz J, Oei A, Földvári G, Hovius J, Takken W, Sprong H. Larvae of *Ixodes ricinus* transmit *Borrelia afzelii* and *B. miyamotoi* to vertebrate hosts. Parasit Vectors. 2016 Feb 20;9:97

3. P4 Line 4,7– The genus name '*Borrelia*' was abbreviated at the beginning of the sentences here and in P17 line 18 and P19 line 25, the genus name '*Borrelia*' was spelled out at the beginning of the sentences. Please be consistent. I suggest spelling out the genus name when it was used at the beginning of the sentence.

   RESPONSE: Abbreviation (B.) was changed to "*Borrelia*" when it was used at the beginning of the sentence at P4 Line 4,7 and throughout the manuscript.

4. P4 Line 6, 9 – again the references, please cite original research articles throughout the manuscript.

   RESPONSE: We are very sorry for the mistake. The original research articles were cited in the manuscript and are listed here as well.

- Fukunaga M, Takahashi Y, Tsuruta Y, Matsushita O, Ralph D, McClelland M, et al. Genetic and phenotypic analysis of *Borrelia miyamotoi* sp. nov., isolated from the ixodid tick *Ixodes persulcatus*, the vector for Lyme disease in Japan. Int J Syst Bacteriol. 1995;45(4):804-10.
- Scoles GA, Papero M, Beati L, Fish D. A relapsing fever group spirochete transmitted by *Ixodes scapularis* ticks. Vector Borne Zoonotic Dis. 2001;1(1):21-34.
- Fraenkel CJ, Garpmo U, Berglund J. Determination of novel *Borrelia* genospecies in Swedish *Ixodes ricinus* ticks. J Clin Microbiol. 2002;40(9):3308-12.
- Ogden NH, Margos G, Aanensen DM, Drebot MA, Feil EJ, Hanincova K, et al. Investigation of genotypes of *Borrelia burgdorferi* in *Ixodes scapularis* ticks collected during surveillance in Canada. Appl Environ Microbiol. 2011;77(10):3244-54.

5. P2 Line 7 – Through the manuscript, all the infection rates of *Borrelia* spp. in ticks were estimated from pooled samples. They should be converted to the infection rates of individual tick level such as Minimal Infection Rate (MIR) or other appropriate units otherwise the reported infection rates could be inflated as if all the ticks in the positive pool are infected.

   RESPONSE: The Bias-corrected maximum likelihood estimation (MLE) with lower and upper limits shown in parentheses were added in Table 1. The MLE and MIR estimates were conducted in Excel with the use of the CDC's Mosquito Surveillance Software tool (https://www.cdc.gov/westnile/resourcepages/mosqSurvSoft.html)

6. P4 Line 9 – The cited reference on this sentence is review paper. The original article that reported 3 distinct phylogenetic clades of *B. miyamotoi* should be cited here. (Cosson et al. 2014)

   RESPONSE: The reference of Cosson et al. was cited as recommended in P4 line 9.

- Cosson JF, Michelet L, Chotte J, Le Naour E, Cote M, Devillers E, Poulle ML, Huet D, Galan M, Geller J, Moutailler S, Vayssier-Taussat M. Genetic characterization of the human

relapsing fever spirochete *Borrelia miyamotoi* in vectors and animal reservoirs of Lyme disease spirochetes in France. Parasit Vectors. 2014 May 20;7:233. doi: 10.1186/1756-3305-7-233. PMID: 24886071; PMCID: PMC4039308.

7. P4 Line 11 – I don't think that Fukunaga et al. 1995 and Scoles et al. 2001 confirmed that *Apodemus argenteus* and *Peromyscus leucopus* are the reservoir host of *B. miyamotoi*. Please provide better reference to support this sentence if the authors really think that those two rodent species were confirmed as the reservoir hosts of *B. miyamotoi*, or rephrase the sentence to explain the reservoir host status of those rodent species as described in the cited references. In addition, if the authors want to report any evidence regarding the reservoir hosts of *B. miyamotoi*, then I suggest adding the description or definition of reservoir host of tick-borne pathogen in the manuscript as described by Kahl et al. 2002.

   RESPONSE: The sentence has been revised as "Rodents could play important roles as reservoir hosts and several species have been reported to carry *B. miyamotoi*. For example, *Apodemus argenteus* from Japan (13) and *Peromyscus leucopus* from USA (10) were found to be naturally infected with the bacterium while *Myodes glareolus* from France (18), *Apodemus flavicollis* from Hungary (19) were likely to play a role in the transmission cycle as well." P4 line 9-14.

   Moreover, the definition of reservoir host has been added into the discussion part (P19 lines 6-14) "However according to Kahl et al.(27), the definition of the reservoir host and vector in the vector-borne transmission requires three key components 1) the pathogen has to be transmitted to the reservoir host via a vector bite, 2) pathogen must survive and multiply in the reservoir host, and 3) the pathogen must subsequently be acquired by a new vector upon biting the reservoir host and be transmitted to further hosts. The detection of *B. miyamotoi* in *I. granulatous* ticks collected from rodents in this study may or may not imply that tick or rodent species are vector or reservoir hosts for *B. miyamotoi*. In order to make this conclusion, studies of vector competence and reservoir host competence must be carried out experimentally."

8. P4 Line 14 – What facts in the sentence are supported by Ref.10?

   RESPONSE: We moved the reference and placed at the end of this statement. *B. miyamotoi* was detected in several rodent species (*Bandicota indica*, *Mus caroli*, and *Niviventer tenaster*) in Thailand (ref; Takhampunya et al. 2021), there has not yet been any study performed to confirm their roles as reservoir hosts in the maintenance of the bacterium in nature.

9. P5 Line 24 – Change the subtitle of this paragraph since it also includes trapping methods.

   RESPONSE: The subtitle has been changed to "**Rodent trapping and tick collection**".

10. P5 Line 29-31 – What types of traps were used? Sherman trap? It might be helpful to better understand the sampling process if the authors provide more details of field sampling such as what types of trap? how many traps used? How the placement of the traps were arranged?

RESPONSE: The live traps were used in this study. The detail of rodent trapping below was added in the method section (P5 lines 31-32 and P6 line1-5) under the subtitle **Rodent trapping and tick collection**.

"All traps were set up in the evening between 1900 and 2200, and checked early in the morning. Traps were set for 3–5 nights for each collection. Traps were placed between 100-120 traps per night in the format of either grid or transect according to the topography. Trapping was organized at each collection site using several trap-lines and help from locals or the owner of the field for the information on the presence and the abundance of rats, their tracks, and locations of burrows in the edges of fields."

11. P6 Line 23, 26 and P7 line 2,3 – typo on the symbol of degree-Celsius. Remove space between degree symbol and Celsius symbol.

    RESPONSE: The space was removed between degree and Celsius symbols.

12. P9 Line 14 – Personally, I was a bit surprised from the result that only 43 ticks were collected from 640 captured animals and even there was no *Ixodes* larva. This result was not consistent from the previous report of the authors' group, Takhampunya et al. 2021. Author's opinion or explanation about this small number of ticks collected from captured animals and absence of *Ixodes* larva may help the readers to better understand the disease ecological feature of the vector ticks for *B. miyamotoi* in Thailand.

    RESPONSE: Both publications authored by Takhampunya et al. 2021 did not find larva of *Ixodes* spp. either. The results of the retrospective study where previous collections were screened for *Borrelia* spp. was also consistent with this current study where only small number of ticks (N=6) were collected from animals captured in Tak province (N=305). We did try several times and several sites in Phop Phra district to collect questing ticks by dragging & flagging from several locations including the rodent trapping sites, there was no questing tick present. Unlike other sites in Thailand where we successfully collected questing ticks from forest areas or even orchards, edges of dense forest, or national park (Khao Yai national park). But in this particular area in Phop Phra district where the majority of populations (85%) are farmers (maze, rice, vegetables, and legumes), the use of herbicide and pesticide as well as the agricultural burning in preparation for the next crop cycle might be somehow attributed to the small number of ticks collected from this area. However, this is only my personal opinion.

    The following statement was added in the discussion part (P19 lines 23-28) "Only a small number of ticks was collected from rodents in this area and attempts to collect questing ticks by dragging and flagging at several locations in this area including the rodent trapping sites were unsuccessful. More studies or surveillance should be conducted in other areas in which *B. miyamotoi* was detected such as Nan, Chiangrai, and Loei provinces (Takhampunya et al. 2021)."

13. P10 Line 7 – Since there are large variation on the sample sizes among animal species, I suggest adding confidence interval on each infection rate and seroprevalence rate.

    RESPONSE: Confidence intervals were added for the infection rate and the % serological reactivity in Table 1, 3, and 5 as well as in the text for the % serological reactivity in humans.

14. P10 Line 11– It would be nice if the authors explain whether the positive ticks were collected from positive animals or not.

    RESPONSE: No positive ticks were collected from animals with PCR-positive *Borrelia* spp. However, one *B. yangtzensis*-positive *Haemaphysalis* tick (nymph) were collected from *Rattus tanezumi* that had serological reactivity with recombinant GlpQ protein of *B. miyamotoi* (titer 800) and had PCR-negative result for *Borrelia* spp. This following information was also added in the discussion part (P19 Lines 21-23).
    "No *Borrelia*-positive tick was collected from *Borrelia*-positive animals in this study. However, the detection of *Borrelia* spp. in feeding ticks does not necessarily imply the vectorial status of the tick species."

15. P11 Line 1, Table 1. Typo. '*Rattus exultans'* should be '*Rattus exulans'*. Please describe what animal species are in 'Others' category from trapped animals.

    RESPONSE: The typo has been corrected to *Rattus exulans* in the Table 1. For other species, the full list of other species was provided under the Table 1 as follows;

    Other species = *Berylmys mackenziei*, *Menetes berdmorei*, *Niviventer fulvescens*, *Tupaia belangeri*, *Vandeleuria oleracea*

16. P19 Line 1-3 – Again, the authors should use the term 'Reservoir host' more carefully. The cited reference (Ref. 10) just reported finding of *B. miyamotoi* DNA from the animal species listed in the sentence. This finding doesn't prove that the positive animal species are 'reservoir host' of *B. miyamotoi*. They could be 'potential' reservoir hosts. Please rephrase the sentence.

    RESPONSE: We agree with the reviewer and the word 'reservoirs host" was deleted from the sentence and replaced with "several rodent species".
    P18 line 21 and P19 lines 1-3, added "Its distribution in other regions of the country was later reported in the retrospective study, which revealed its detection in several rodent species (*Bandicota indica*, *Mus caroli*, *Rattus surifer,* as well as *Niviventer tenaster*) which could potentially play a role in maintaining *B. miyamotoi* enzootic life cycle (19)."

17. P19 Line 18 – This study didn't find *B. miyamotoi* infection in humans as mentioned in line 18. This sentence can be deleted since the authors expressed same meaning at the sentence in line 28 – 30.

    RESPONSE: The sentence was deleted as recommended (P19 line 4). The paragraph has been revised as follows (P19 Lines 18-21);
    "Although *B. miyamotoi* was not detected in human samples by molecular methods, the serological reactivity of human and rodent IgG antibodies against rGlpQ protein of *B. miyamotoi* along with the detection of spirochete bacteria in rodents and ticks is a strong indication of *B. miyamotoi* transmission in this area."

18. P19 Line 4 – Same as 'reservoir host', this study just found *I. granulatus* infected with *B. miyamotoi* collected from wild animals. It doesn't prove *Ixodes granulatus* as the vector for *B. miyamotoi.* Please rephrase the sentence.

    RESPONSE: The paragraph has been rephrased to read (P19 Lines 4-14) "This study is the first detection of *B. miyamotoi* in tick species, *Ixodes granulatus,* indicating this species could play a role in maintaining the enzootic transmission cycle of *B. miyamotoi* in nature. However according to Kahl et al.(27), the definition of the reservoir host and vector in the vector-borne transmission requires three key components 1) the pathogen has to be transmitted to the reservoir host via a vector bite, 2) pathogen must survive and multiply in the reservoir host, and 3) the pathogen must subsequently be acquired by a new vector upon biting the reservoir host and be transmitted to further hosts. The detection of *B. miyamotoi* in *I. granulatous* ticks collected from rodents in this study may or may not imply that tick or rodent species are vector or reservoir hosts for *B. miyamotoi*. In order to make this conclusion, studies of vector competence and reservoir host competence must be carried out experimentally."

19. P20 Line 27 – typo. 'areassuch as'
    RESPONSE: The typo was correct as "areas such as".

20. P20 line 34-35 and P21 Line 1-2– Again, the detection of the bacteria from the animal species and ticks didn't prove the animals or the ticks are 'reservoir' or 'vector', respectively. Please rephrase the sentence.

    RESPONSE: Words "reservoir" and vector are deleted from the sentence.

21. P21 Line 4-5 – If there is a reference that reported the vector competence of *Ixodes granulatus* for *B. miyamotoi*, please cite the reference here. Otherwise, vector competence of *I. granulatus* for *B. miyamotoi* should be investigated first.

    RESPONSE: There is no study regarding the vector competency of *Ixodes granulatus* for *B. miyamotoi* infection and transmission. Although, *Ixodes granulatus* is the predominant *Ixodes* species collected from rodents in Thailand and it is a member of *I. ricinus/I. persulcatus* species complex (reference below), which are confirmed vectors of *B. miyamotoi*. No one has ever studied its ability to transmit this spirochete bacterium. Therefore, sentences and wordings that misled the readers to believe that *I. granulatus* is the vector for *B. miyamotoi* have been revised throughout the manuscript. P21 Lines 4-5, sentence has been revised as "The possibility that *I. granulatus* or other species may serve as vectors should be explored."

    - Xu, G., Fang, Q.Q., Keirans, J.E. & Durden, L.A. (2003) Molecular phylogenetic analyses indicate that the *Ixodes ricinus* complex is a paraphyletic group. Journal of Parasitology, 89, 452–457. https://doi.org/10.1645/0022-3395(2003)089[0452:MPAITT]2.0.CO;2

22. P21 Line 8 – Please cite the reference that supports the high density of *I. granulatus* in mountainous areas.

RESPONSE: The sentence has been revised to read (P21 lines 5-8) “More work is required to accurately evaluate the prevalence, range, and risk of human exposure, especially in areas such as forest habitats, mountain ranges, national park where *I. granulatus* is found predominantly (ref).

Four articles (shown below) were cited after this sentence. Although, they did not stated in their articles that the high density of *Ixodes granulatus* was high in the mountainous area, the collection sites and areas (provinces) where *I. granulatus* was collected are mostly in the high land or mountain ranges such as Nan, Loei, Chiangmai, Tak, Nakhon nayok, Nakhon ratchasrima, or areas near the national park. Guglielmone et al. and Petney et al. published an extensive review of tick species in the world and Southeast Asia, respectively and they stated that *I. granulatus* is found predominantly in forest habitats in a variety of ecoregions.

- Petney TN, Saijuntha W, Boulanger N, Chitimia-Dobler L, Pfeffer M, Eamudomkarn C, et al. Ticks (Argasidae, Ixodidae) and tick-borne diseases of continental Southeast Asia. Zootaxa. 2019;4558(1):1-89.
- Cornet J-P, Demoraes F, Souris M, Kittayapong P, Gonzalez J-P. Spatial distribution of ticks in Thailand: a discussion basis for tick-borne virus spread assessment. International Journal of Geoinformatics. 2009;5.
- Tanskul P, Stark HE, Inlao I. A checklist of ticks of Thailand (Acari: Metastigmata: Ixodoidea). J Med Entomol. 1983;20(3):330-41.
- Guglielmone AA, Robbins, R.G., Apanaskevich, D.A., Petney, T.N., Estrada-Peña, A. & Horak, I.G. The Hard Ticks of the World (Acari: Ixodida: Ixodidae). Heidelberg: Springer; 2014. xiii+738 p.

Reviewer #1:
General comments

Major comments
The study is complex and aimed to evaluate the borrelia spp. species in a one health approach. The manuscript in general is well written and structured. However, some aspects are omitted and rise important questions/concerns. Few are presented below:

1. It is not clear if/how the sensibility and more important the specificity of the in-house ELISA assay was evaluated;

   RESPONSE: Since there is no commercial kit or positive serum control available on the market, we had tried to reach out to some labs but received no response. Therefore, the only option for us was to establish an in-house ELISA assay by following previously published protocols (references below).The references have been added in the method section as well. We also confirmed the specific binding between antigen (rGlpQ protein) and IgG antibody from patient serum that had serological reactivity to rGlpQ with a titer of 400 using a western blot assay and showed that only a positive sample (not a negative one) produced a signal at an expected protein size (38 KDa) as shown in Fig 4B. We also added the results of the cross-reactivity of the in-house ELISA assay in this manuscript as well (Table 3). The cross-reactivity test was performed against vector-borne diseases previously reported in Thailand such as scrub typhus, murine

typhus, anaplasmosis, bartonellosis, and spotted fever *Rickettsia. Borrelia burgdorferi* was included in this test in order to determine the cross-reactivity of the in-house ELISA with Lyme *Borrelia*. The results of the cross-reactivity has been added under the subtitle **Rodent exposure rate to *B. miyamotoi* and evidence of serological reactivity in humans** in the result section (P15 lines 16-26) as follows;
"Furthermore, the cross-reactivity of the in-house ELISA assay was assessed against vector-borne diseases previously reported in the country (24) (Table 3). Positive controls (human IgG antibodies) from available commercial kits were used in this assessment. The results showed that at the initial serum dilution of 1:100 almost all positive controls had OD405 values less than the cutoff value (OD405 = 0.374) with the exception of a positive control for *Anaplasma phagocytophilum* (OD405 = 0.601 $\pm$ 0.091). The positive control of *A. phagocytophilum* was further diluted (1:200-1:3200) and tested with the in-house ELISA assay again. The results showed that the positive control of *A. phagocytophilum* showed cross-reactivity to *B. miyamotoi* rGlpQ protein at the dilution of 1:100; however, the OD value was less than the cutoff value when it was diluted to 1:200 and the cross-reactivity was disappeared at dilutions of 1:400 to 1:3200."

Moreover, this study was initially conducted without any information on human borreliosis cases in the country. Human borreliosis has not been recognized in the country before; therefore, no information is available on the National Disease Surveillance website (Bureau of Epidemiology, Department of Disease Control, MoPH, Thailand) or at the local hospital. This study intended to determine if there is any serological reactivity in the human population as evidence of human exposure to this bacterium. Moreover, patient samples were received from the hospital under non-human subject research protocol, which means we only received the leftover samples from the routine testing at the hospital laboratory. Samples were from patients of undifferentiated fever (fever >38°C) and there was no other human clinical data associated with the samples.

References;

- Krause PJ, Narasimhan S, Wormser GP, Barbour AG, Platonov AE, Brancato J, et al. *Borrelia miyamotoi* sensu lato seroreactivity and seroprevalence in the northeastern United States. Emerg Infect Dis. 2014;20(7):1183-90.
- Wagemakers A, Koetsveld J, Narasimhan S, Wickel M, Deponte K, Bleijlevens B, et al. Variable Major Proteins as Targets for Specific Antibodies against *Borrelia miyamotoi*. J Immunol. 2016;196(10):4185-95.

2. would be interesting to present the associations and or agreements between some results, for example:

   a) if positive ticks were collected from positive hosts (same pathogen involved)?

      RESPONSE: No positive ticks was collected from positive animals as responded previously.

   b) there were cases of PCR positive and seropositive rodent individuals?

RESPONSE: There is no rodent that had both *B. miyamotoi* PCR-positive and seropositive in this study. However for two *B. miyamotoi*-seropositive rodents, one rodent was *B. yangtzensis* PCR-positive and another rodent was infested with *B. yangtzensis*-positive tick.

3. at some point is specified that also ticks from other animals were collected? why did not differentiated the results by tick's vertebrate host? it is any statistically difference?

   RESPONSE: We apologize for the mistake. In this study, no ticks from other domestic animals were collected; only ticks from rodents were investigated.

4. The statistical analysis is missing;

   RESPONSE: The MLE was added in the "Data analysis" subtitle in the methods section (P9 lines 1-4).

<u>Minor comments</u>

1. Avoid personal nouns and abbreviation at the beginning of the sentence (through all the manuscript).

   RESPONSE: Abbreviation at the beginning of the sentence was spell out in full. Personal nouns were removed and the sentences have been revised throughout the manuscript.

<u>Specific comments</u>:

1. P2 line 14: In addition, seroprevalence of *B. miyamotoi* in human samples received from Phop Phra hospital, Tak province and rodents captured from Phop Phra was evaluated using a district using an in-house, direct enzyme-linked immunosorbent assay (ELISA) assay with *B. miyamotoi* glycerophosphodiester-phosphodiesterase (GlpQ) recombinant protein as coated antigen.

   RESPONSE: The sentence has been revised to correct it. "Further investigation was conducted to determine the serological reactivity to *B. miyamotoi* in human samples received from Phop Phra hospital, Tak province and in rodents captured from Phop Phra district using an in-house, direct enzyme-linked immunosorbent assay (ELISA) assay with *B. miyamotoi* recombinant glycerophosphodiester-phosphodiesterase (rGlpQ) protein as coated antigen."

Reviewer #2:

- General Summary: Understanding geographic range, transmission cycle, comparative prevalence, genetic diversity (or lack thereof) and characterizing human infections of *Borrelia miyamotoi* are all topics worthy of research and publication. In this light, I read this manuscript that focuses on *B. miyamotoi* surveillance in Thailand with interest. This team of researchers in Thailand have trapped several genera of rodents (total animals n=640) from a one province (Tak Province), removed ticks off these rodents and tested both rodent tissue (spleen and kidney) and ticks for *B. miyamotoi* by PCR. In addition, they developed a rudimentary serological assay and tested sera collected from a blinded panel of febrile hospitalized patients.

All said, while the topic is interesting, the methodology used for this study has serious errors and thus the results and conclusions are at best, not convincing and at worst, are irresponsible– thus I recommend this manuscript not be accepted for publication in its current form.

Of most concern is the serological assay developed to test human samples. The description of this assay did not list the controls used (it would be recommended to develop using sera from patients who were positive for many different relapsing febrile illnesses (e.g., *Borrelia hermsii*, *B. duttoni*…as well as from patients positive for *Borrelia* spp. illnesses (e.g., *Borrelia burgdorferi* or *Borrelia mayonii*).

Also, since these patients had acute infections as they were in the hospital, it would be appropriate to test by PCR in the case they hadn't yet been able to mount an immune reaction. Note: the authors did test the patients sera by PCR and all sera samples tested negative but this result was really hidden in the manuscript.

Secondly, to validate a serological assay, patients should have been screened for history of tick bite as well as relapsing fever and presented onset date and sera collection date (acute and chronic sera collection). At this point, it is irresponsible to state a 17.9% seroprevalence in humans in this study population using this non-validated serological assay. I would remove any mention of human testing from future papers unless this work is revised and all testing conducted appropriately for development of a human diagnostic assay with reporting of sensitivity and specificity.

RESPONSE: The *Borrelia* detection by molecular assay in humans was added in the results section (P10 lines 20-21) "All human samples (N=84) were negative for a genus-based TaqMan real-time PCR assay targeting the *Borrelia* 16S rRNA gene (S1 Table)." The supporting information S1 Table was submitted along with the revised manuscript and indicated in P21 lines 10-13.

As mentioned earlier in responses to reviewer#1's comments, the in-house ELISA assay used in this study was not intended to be used as a human diagnostic assay but rather to investigate the human exposure to the bacterium, *Borrelia miyamotoi*. Since there is no commercial kit or positive human serum available, a purified recombinant GlpQ protein was purchased from the company and used as a coated antigen in the in-house ELISA assay for IgG antibody detection in rodents and humans by following the instructions previously published by other groups (please find the references below). The cross-reactivity of the in-house ELISA assay was added in this manuscript (Table 3). The cross-reactivity test was performed against vector-borne diseases previously reported in Thailand such as scrub typhus, murine typhus, anaplasmosis, bartonellosis, and spotted fever *Rickettsia*. *Borrelia burgdorferi* was included to determine the cross-reactivity of the in-house ELISA with Lyme *Borrelia*. The results of cross-reactivity was also added in the result section (P15 lines 16-26).

Moreover, the manuscript has been revised to remove any statement or determination regarding human infection from the results obtained from the in-house ELISA assay in this study. The below paragraph was added in the discussion section (P19 lines 30-37 and P20 lines 1-3). "However, the cross-reaction to *B. miyamotoi* rGlpQ protein was observed with the positive control (human IgG) of *A. phagocytophilum* at the dilution of 1:100 but the cross-reactivity disappeared when it was diluted further (1:200-1:3200). Similar observation was reported by

Jahfari et al. in the serologically unconfirmed but suspected human granulocytic anaplasmosis in the Netherlands (8). Moreover, other groups also observed the low sensitivity and specificity of *B. miyamotoi* rGlpQ protein and suggested using other variable major proteins (Vmps) in conjunction with rGlpQ protein to improve serodiagnostic sensitivity (28, 29). In this current study, four human samples with high IgG antibody titer (400) were detected and the specific antigen-antibody binding was later confirmed by the Western blot assay. Potentially, the data presented in this study provide the serological evidence of *B. miyamotoi* exposure in humans in Thailand."

References;

- Wagemakers A, Koetsveld J, Narasimhan S, Wickel M, Deponte K, Bleijlevens B, et al. Variable Major Proteins as Targets for Specific Antibodies against *Borrelia miyamotoi*. J Immunol. 2016 May 15;196(10):4185-95.
- Krause PJ, Narasimhan S, Wormser GP, Barbour AG, Platonov AE, Brancato J, et al. *Borrelia miyamotoi* sensu lato seroreactivity and seroprevalence in the northeastern United States. Emerg Infect Dis. 2014 Jul;20(7):1183-90.

❖ The rodent and tick surveillance portion of this study also have serious errors and assumptions, most glaring is testing ticks removed from animals and assuming that these are infected with *B. miyamotoi* or act as possible vectors. While these ticks may harbor *B.miyamotoi*-infected blood acquired from their host, they may not able to sustain infection or transmit. To characterize the prevalence in ticks, unfed ticks must be collected from the environment prior to testing.

RESPONSE: The statements regarding reservoir hosts or vector for *B. miyamotoi* in this manuscript have been revised as in the responses to comments above and in the discussion part (P19 lines 4-14). Moreover, the definition of reservoir host and vector has been added in the discussion part (P19 lines 6-14).

❖ Minor issues that should be addressed include the definition of reservoir and vector: just finding a rodent positive does not make it clear it is a reservoir and finding a tick off an animal does not define the species as a vector: vector and host competence studies need to be conducted first. Nevertheless, it is a great first step in clarifying the transmission cycle in a region and I hope these researchers continue to move forward with additional studies on *Borrelia miyamotoi* in Thailand.

RESPONSE: We have addressed all comments above. The reservoir host and vector definition for the pathogen was provided in the discussion section as well as in the response to the above comments. We will continue working on tick-borne disease in Thailand including understanding the disease epidemiology in the region.

Reviewer #3:

The manuscript describes informative findings regarding the eco-epidemiological feature of *B. miyamotoi* in Thailand. This report will improve our knowledge about the disease ecology and geographical distribution of *B. miyamotoi*. I appreciate that the authors provide serological evidence of *B. miyamotoi*

infection in humans. The presentation needs improvement. The referencing is weak, missing several key references.

RESPONSE: Thank you for your comments. Key references have been added and revised in the manuscript. This is the first attempt to establish and investigate the serological reactivity to *B. miyamotoi* in human and rodent populations. As commercial kit is not available for *B. miyamotoi* and the only option we have is to establish the in-house assay using commercial available recombinant protein from the outsource company and by following previously published protocols.
