# Supplementary material for: Borrelia miyamotoi a neglected tick-borne relapsing fever spirochete in Thailand
Source: PLoS Negl Trop Dis. 2023 Feb 21;17(2):e0011159. doi: 10.1371/journal.pntd.0011159 (PMC9983830; doi:10.1371/journal.pntd.0011159)
Supplement: Attachment — Submitted filename: Response Letter_09Feb2023.pdf [file pntd.0011159.s004.pdf]

February 09, 2023

Wen-Ping Guo
Mathieu Picardeau
Academic & Section Editors,
PLOS Neglected Tropical Diseases

Dear Professors Wen-Ping Guo & Mathieu Picardeau:

We would like to submit a 2nd revised version of the manuscript entitled "*Borrelia miyamotoi* a neglected tick-borne relapsing fever spirochete in Thailand" for consideration by the PLOS Neglected Tropical Diseases. Below you will see our responses to the reviewers' comments/questions. The revised manuscript uploaded separately shows those changes to the manuscript made in response to the reviewers' recommendations in a tracked changes file. In addition, the Table 1 and the results part have been revised to include the re-calculation of the point estimate infection rate of MLE and MIR in pooled ticks. Moreover, the sampling years for rodents and humans have been correct throughout the manuscript as well as in a supplementary Table. This manuscript is not currently being submitted to another journal.

Thank you once again for your time and consideration.

Sincerely,

Ratree Takhampunya, Ph.D.
Department of Entomology
Armed forces Research Institute of Medical Sciences
U.S. Army Medical Component
3I5/6 Rajvithi Road
Bangkok 10400, THAILAND
Ratreet.fsn@afrims.org

The response to reviewers' comments/suggestions as the following:

- ❖ Methods

-Are the objectives of the study clearly articulated with a clear testable hypothesis stated?

-Is the study design appropriate to address the stated objectives?

-Is the population clearly described and appropriate for the hypothesis being tested?

-Is the sample size sufficient to ensure adequate power to address the hypothesis being tested?

-Were correct statistical analysis used to support conclusions?

-Are there concerns about ethical or regulatory requirements being met?

Reviewer #3: No major additional analysis is required, but a few minor comments are suggested in Editorial and Data Presentation Modifications.

Response= Thank you. We have revised the results part and the data in Table 1 as recommended.

- ❖ Results

-Does the analysis presented match the analysis plan?

-Are the results clearly and completely presented?

-Are the figures (Tables, Images) of sufficient quality for clarity?

Reviewer #3: No major revision is required, but a few minor comments are suggested in Editorial and Data Presentation Modifications.

Response= The response is provided below.

- ❖ Conclusions

-Are the conclusions supported by the data presented?

-Are the limitations of analysis clearly described?

-Do the authors discuss how these data can be helpful to advance our understanding of the topic under study?

-Is public health relevance addressed?

Reviewer #3: No major revision.

Response= Thank you.

- ❖ Editorial and Data Presentation Modifications?

Use this section for editorial suggestions as well as relatively minor modifications of existing data that would enhance clarity. If the only modifications needed are minor and/or editorial, you may wish to recommend "Minor Revision" or "Accept".

Reviewer #3: 1. Sampling years for rodent trapping and human blood are confusing. Sampling periods were described differently in several places as listed below.

In introduction P4 Line 31-32, sampling years were described as "(2019-2020)" for ticks and rodents and "(2018-2019)" for human patients. But in Materials and Methods, P5 Line 15-16, human sampling period was described as February-November 2017. Line 25, sampling period of rodent and ticks was described as "2019 (Feb thru Dec)". In Results section, in the first subtitle (P9 Line 7-8) and the first paragraph, sampling year for rodents and ticks was described as 2019 only. In the second subtitle of the results and the contents, P10 Line 1-2 and Line 5-6, it looked like sampling year for human, rodents and ticks were 2019-2020. P18 Line 4-5, human sampling period was described as "A total of 84 patient sera were collected from this hospital during 2018-2019." S1 Table, there is a note describing "Samples (human) were collected during February-November 2017."

Please clarify the sampling years for rodents and humans, and be consistent throughout the manuscript.

Response= Thank you and we are very sorry for the mistakes. We have revised the date for rodents and ticks collection to "every other month from February thru December in 2019 and in February, July, and September 2020". For human samples, samples were from outpatients and inpatients presenting to Phrop Phra hospital with UFI during 2018-2019. We did not received the

actual blood collection date and we only have samples receiving date. Therefore, for human samples, we have changed the collection time to during 2018-2019.

2. P9 Line 1-4, this is a very poor sentence. It didn't describe what data were estimated with the statistical method described in the sentence.

The infection prevalence of ticks was estimated from pooled samples, so it was suggested to use any statistical methods for estimating infection rates from pooled samples. Because if not, the infection rates of ticks could be mis-interpreted as if all the ticks in the positive pools were infected. For example, in Abstract at P2, the authors summarized that they collected 43 ticks (line 5) and reported the infection rate of ticks as 22.2% (line 8). This could be misread as about 22.2% of 43 ticks (10 ticks) were infected. But if we use MIR (minimum infection rate) with the result of 6 positive pools from 27 pools of 43 ticks, the MIR of the ticks will be about 13.95% (6 positive ticks from 43 ticks).

The authors responded that they used the Excel add-in program for mosquito surveillance provided by CDC. I am glad that the author used MIR with the CDC program that doesn't need an assumption required in the traditional MIR such as when a pool is positive then only one individual in that pool is positive. To my knowledge, the excel add-in tool will use the bias-corrected MLE for the point estimate infection rate and the skewness-corrected score for CI. The author can just use the point estimation of MIR calculated by bias-corrected MLE and the corrected score CI provided by the add-in tool. There is no need to add a column for MLE limits in the table. The table 1 in the revised manuscript still has the infection rate of ticks calculated by using the number of pools as the denominator. I still think that the infection rate of ticks should be reported from the individual tick number base, not from the pooled group testing.

Response= The MLE and MIR for *Borrelia* infection rate were re-calculated again and added in the Table 1. The infection rate of ticks calculated by using the number of pools as the denominator was revised and only the number of *Borrelia*-positive tick pool (%) was mentioned. The infection rate was also revised in the text (abstract and result parts) as well.

Moreover, the "Data Analysis" section has revised to include the below information;

"To estimate the probability of infection rate in pooled ticks, a maximum likelihood estimation (MLE) and a minimum infection rate (MIR) were calculated in Excel with the use of the CDC's Mosquito Surveillance Software tool

(https://www.cdc.gov/westnile/resourcepages/mosqSurvSoft.html), which calculate the point estimate infection rate and 95% confidence intervals (CI) using pooled data that take into account individual pool samples sizes."

- Summary and General Comments

Use this section to provide overall comments, discuss strengths/weaknesses of the study, novelty, significance, general execution and scholarship. You may also include additional comments for the author, including concerns about dual publication, research ethics, or publication ethics. If requesting major revision, please articulate the new experiments that are needed.

Reviewer #3: The manuscript describes informative findings regarding the eco-epidemiological feature of *B. miyamotoi* in Thailand. This report will improve our knowledge about the disease ecology and geographical distribution of *B. miyamotoi.*

In the revised manuscript, the referencing and the structure of the manuscript were improved, but it still requires a few minor modifications as described at Editorial and Data Presentation Modifications. The authors already have an appropriate tool to re-estimate infection prevalence of ticks from pooled samples, so I believe that the suggestion on reporting infection prevalence of ticks can be done by minor revision.

Response= Thank you for very helpful advice on how to calculate the infection rate for pooled ticks and the correction in other sections of the manuscript. We are grateful for the reviewer's time and attention to improve this manuscript. The calculation was revised as recommended (please see our responses in the above comments).
