# Supplementary material for: Borrelia miyamotoi a neglected tick-borne relapsing fever spirochete in Thailand
Source: PLoS Negl Trop Dis. 2023 Feb 21;17(2):e0011159. doi: 10.1371/journal.pntd.0011159 (PMC9983830; doi:10.1371/journal.pntd.0011159)
Supplement: Attachment — Submitted filename: PLOS NTD 2022 B. miyamotoi in Thailand.docx [file pntd.0011159.s002.docx]

**PLOS NTD**

**MS PNTD-D-22-01143**

***Borrelia miyamotoi* a neglected tick-borne relapsing fever spirochete in Thailand**

**Authors: Takhampunya, R. et al.**

**General Summary:** Understanding geographic range, transmission cycle, comparative prevalence, genetic diversity (or lack thereof) and characterizing human infections of Borrelia miyamotoi are all topics worthy of research and publication. In this light, I read this manuscript that focuses on B. miyamotoi surveillance in Thailand with interest. This team of researchers in Thailand have trapped several genera of rodents (total animals n=640) from a one province (Tak Province), removed ticks off these rodents and tested both rodent tissue (spleen and kidney) and ticks for B. miyamotoi by PCR. In addition, they developed a rudimentary serological assay and tested sera collected from a blinded panel of febrile hospitalized patients.

All said, while the topic is interesting, the methodology used for this study has serious errors and thus the results and conclusions are at best, not convincing and at worst, are irresponsible– thus I recommend this manuscript not be accepted for publication in its current form. Of most concern is the serological assay developed to test human samples. The description of this assay did not list the controls used (it would be recommended to develop using sera from patients who were positive for many different relapsing febrile illnesses (e.g., Borrelia hermsii, B. duttoni…as well as from patients positive for Borrelia spp. illnesses (e.g., Borrelia burgdorferi or Borrelia mayonii). Also, since these patients had acute infections as they were in the hospital, it would be appropriate to test by PCR in the case they hadn’t yet been able to mount an immune reaction. Note: the authors did test the patients sera by PCR and all sera samples tested negative but this result was really hidden in the manuscript. Secondly, to validate a serological assay, patients should have been screened for history of tick bite as well as relapsing fever and presented onset date and sera collection date (acute and chronic sera collection). At this point, it is irresponsible to state a 17.9% seroprevalence in humans in this study population using this non-validated serological assay. I would remove any mention of human testing from future papers unless this work is revised and all testing conducted appropriately for development of a human diagnostic assay with reporting of sensitivity and specificity.

The rodent and tick surveillance portion of this study also have serious errors and assumptions, most glaring is testing ticks removed from animals and assuming that these are infected with B. miyamotoi or act as possible vectors. While these ticks may harbor B.miyamotoi-infected blood acquired from their host, they may not able to sustain infection or transmit. To characterize the prevalence in ticks, unfed ticks must be collected from the environment prior to testing.

Minor issues that should be addressed include the definition of reservoir and vector: just finding a rodent positive does not make it clear it is a reservoir and finding a tick off an animal does not define the species as a vector: vector and host competence studies need to be conducted first. Nevertheless, it is a great first step in clarifying the transmission cycle in a region and I hope these researchers continue to move forward with additional studies on Borrelia miyamotoi in Thailand.
